# Tissue Engineering Strategies for Peripheral Nerve Regeneration

**DOI:** 10.3389/fneur.2021.768267

**Published:** 2021-11-16

**Authors:** Yin Li, Zhenjiang Ma, Ya Ren, Dezhi Lu, Tao Li, Wentao Li, Jinwu Wang, Hui Ma, Jie Zhao

**Affiliations:** ^1^Shanghai Key Laboratory of Orthopaedic Implant, Department of Orthopaedic Surgery, Shanghai Ninth People's Hospital, Shanghai Jiao Tong University School of Medicine, Shanghai, China; ^2^Southwest JiaoTong University College of Medicine, Chengdu, China; ^3^School of Medicine, Shanghai University, Shanghai, China; ^4^Department of Orthopaedics, Xinhua Hospital Affiliated to Shanghai Jiaotong University School of Medicine, Shanghai, China

**Keywords:** peripheral nerve regeneration, nerve tissue engineering, pathophysiology, scaffolds, cells, 3D printing

## Abstract

A peripheral nerve injury (PNI) has severe and profound effects on the life of a patient. The therapeutic approach remains one of the most challenging clinical problems. In recent years, many constructive nerve regeneration schemes are proposed at home and abroad. Nerve tissue engineering plays an important role. It develops an ideal nerve substitute called artificial nerve. Given the complexity of nerve regeneration, this review summarizes the pathophysiology and tissue-engineered repairing strategies of the PNI. Moreover, we discussed the scaffolds and seed cells for neural tissue engineering. Furthermore, we have emphasized the role of 3D printing in tissue engineering.

## Introduction

A peripheral nerve injury (PNI) is a medical problem mainly caused by external trauma after stretching, tearing, or extrusion of peripheral nerves. (1) It has attracted social attention because of its enormous social and economic pressure. In the United States, the economic loss caused by nerve injury exceeds $150 billion annually, and the treatment cost exceeds billions of dollars every year. People often underestimate the incidence of PNI, which seriously affects the quality of life of the patients (1–3).

The peripheral nervous system can self-regenerate and repair itself after injury. However, the repair is often slow and incomplete because axon extension depends on the synthesis and transportation of the intracellular substances, and the regeneration speed is similar to that of axon transportation, about 1–3 mm/day (4). In general, the stretch or crush injury results are better than those of transection. The recovery of the distal injury is better than that of the proximal injury, because axon growth only needs a short distance to reach the distal stump.

In the previous studies, substantial functional recovery of mild and moderate nerve injury can be achieved by surgical operation (such as, nerve suture and nerve transplantation) or non-surgical operation (such as, magnetic field, electric field, He-Ne Laser, and traditional Chinese Medicine) (3). The nerve suture and nerve grafts are extensively used for surgical nerve repair in the experiment and the clinic. An end-to-end nerve suture requires that the nerve stumps are aligned to achieve the best repair effect, and only the short nerve gap (<5 mm) can be used (5). When the damage gap is more significant (more than 5 mm), it is not possibly repaired by the meticulous microscopic surgery, and nerve autograft is regarded as the gold standard (3, 5). However, this damages the healthy nerves, and the number of donors is limited (6).

With the rapid development of cell biology and materials science, a new discipline, tissue engineering, is established to construct a different method of peripheral nerve repair. The core of nerve tissue engineering is to build a three-dimensional complex composed of cells and biomaterials and make the nerve guiding catheters (NGCs). The catheters are active scaffolds that can effectively guide axon regeneration. They contain the essential cells and neurotrophic factors that support axon regeneration. Implantation of the NGCs in the injured site can simulate the neural structure after Wallerian degeneration and avoid immune reactions. Even in some injury models, the clinical effect is similar to that of autologous nerve transplantation. The ideal NGCs should have excellent mechanical strength, good biocompatibility, biodegradability, and permeability (7).

In recent years, the research on neural tissue engineering has become more in-depth, and various NGCs have emerged one after another. To find a more suitable clinical treatment, the researchers need to pay attention to the regeneration effect of axons and focus on the transformation of basic research to clinical. Here, we aimed to review the pathophysiology and repairing strategies for PNI, focusing on the latest advances in nerve tissue engineering.

## Pathophysiology

### Anatomical Physiology

The periphery nerve system consists of all the nerves from the three main categories: the spine nerve, cranial nerve, visceral nerve, and their associated ganglia (8). The first two types of nerve fibers with myelin sheath are Schwann cells (SCs) wrapped in several layers, while the third nerve fibers are generally non-myelinated. The nerve bundles include the nerve fibers and connective tissue, which constitute the nerves as mentioned above. A connective tissue can be divided into three layers: epineurium, perineurium, and endoneurium. It contains the blood vessels in the epineurium, which provide the nerve fibers with trophic support. In the endoneurium, there are different types of cells except for connective tissue and nerve fibers (9, 10). For example, SCs produce neurotrophins and extracellular matrix (ECM), thus providing the nerve-growth microenvironment. In addition, it plays a crucial role in nerve regeneration. The macrophages are the essential cells in the inflammatory response, generally divided into two phenotypes and play different roles in nerve degeneration and regeneration (11).

### Injury Types

Many factors contributed to the PNI (12). The basic injury types seen in the clinical practice are three main kinds (Table 1). The most common type is stretch-related injury. As we all know, after an external force pulls the spring, the spring will stretch in the direction of the power, and when the intensity exceeds a specific limit, plastic deformation will occur. Similarly, an injury occurs when traction forces exceed the stretch limit strength of the nerves, whose properties are similar to the properties of spring (15). For example, excessive traction caused by an overextension of the neck at birth leads to a particular type of birth paralysis limited to the fifth and sixth cervical nerves, which is also called Erb's palsy (13).

**Table 1:** Common types of nerve damage.

**Injury type**	**Continuity**	**Injury mechanism**	**Recovery speed**	**Example**	**References**
Stretch-related injury	Generally keep	Stretching force exceeds the elastic limit of the nerve membrane.		Erb's palsy	(13)
Laceration	Mostly keep	The nerve membrane is wholly or partially ruptured.	2–3 mm/day	Common, such as knife wounds	(12)
Compression	Generally keep	Nerve compression or prolonged ischemia (>8 h).	3–4 mm/day	Radial neuropathy	(14)

The laceration is another essential type of PNI, accounting for about 30% of the serious injuries (12). In this case, these can be complete transection of the nerve, and hence the process of nerve degeneration and regeneration can be observed clearly. Compression is the third injury type. There are two mechanisms of the damaged nerve, ischemia and extrusion.

### Injury Scale (IS)

**Figure 1:**
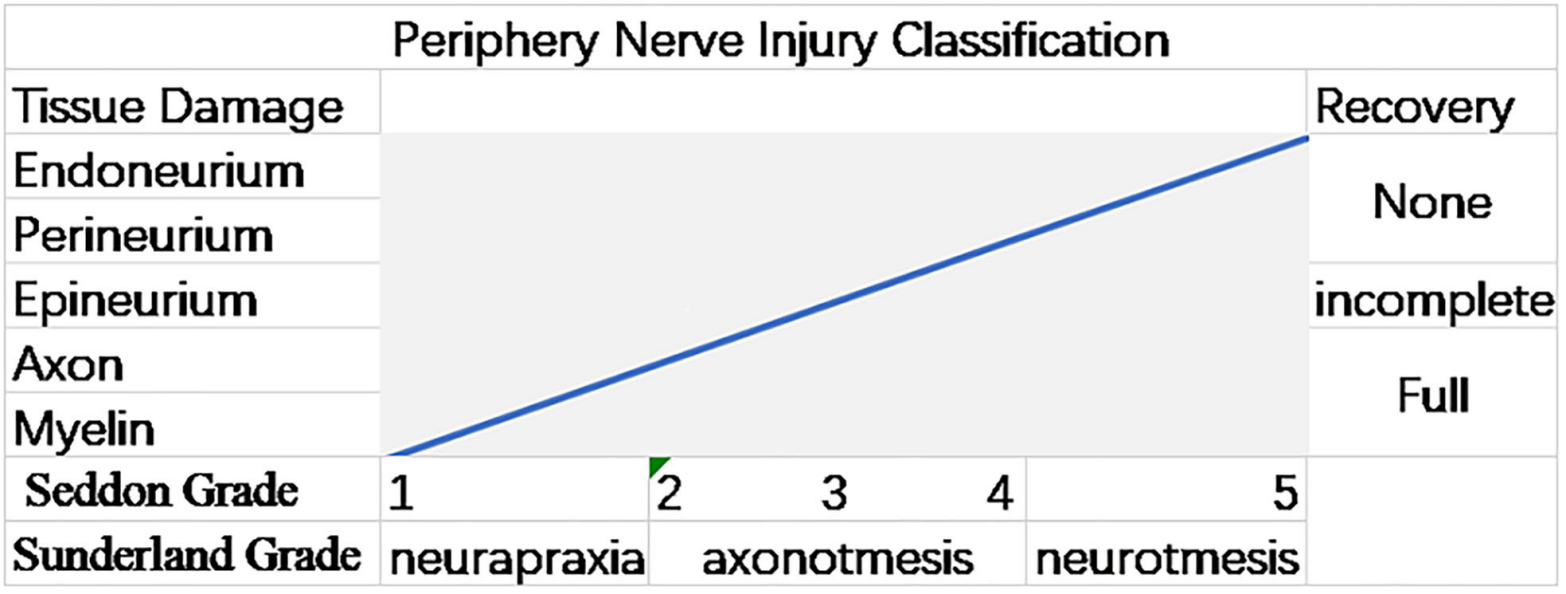
Graph illustrating the Sunderland and Seddon peripheral nerve injury (PNI) grading systems. The gradations in both the systems are associated with the injury's anatomical extent and the chance of a spontaneous (12).

In the clinical diagnosis and treatment of PNI, the first step is to clarify the degree of injury. The most classic injury classifications were proposed by Seddon and Sunderland and are still in use today (Figure 1) (15–17).

The nerve injuries are divided into three categories by Seddon (16). In neurapraxia, the nerve fiber is slightly squeezed, but the axon is not broken, and there is no morphological change, only temporary functional changes. In axonotmesis, the nerve fibers are severely crushed, the axons are broken, and their functions temporarily disappear, accompanied by muscle atrophy, but the myelin and SCs still exist. In neurotmesis, the nerve bundle is broken due to severe trauma, which is difficult to recover. Sometimes it can be recovered from surgery, such as severe laceration. On this basis, Sunderland subdivided the nerve injury classification into five levels (15). The first-degree injury corresponds to Seddon's classification of neuropraxia. From first degree to fifth degree, the severity of the injury increases. The second, third, and fourth-degree injuries correspond to the endoneurium, perineurium, and epineurium lesions. The fifth degree of injury corresponds with the neurotmesis of Seddon, which is irrecoverable damage (3, 12).

## Recovery of Function After Trauma

### Pathological Mechanism

The normal surrounding myelinated nerve is wrapped by a Schwann cell membrane and forms an onion skin-like myelin sheath. This remarkable structure ensures the transmission of specific electrical signals and maintains the normal neurophysiological functions. When an external force acts to stretch, compress, or tear the peripheral nerve, it will cause the distal nerve stump to degenerate, called Wallerian degeneration. At the site of nerve injury, the axon is broken and disintegrated by an external force, forming an inflammatory environment. The myelin sheath is also broken down and lost, and the macrophages are recruited to the injury site to swallow most myelin debris and lipid droplets.

Schwann cells are the most important cells for peripheral nerve regeneration. Due to the transection of the nerve fibers, they lose physical contact with the neuronal cells. Under the combined action of various cytokines (such as transcription factor c-Jun, and histone deacetylase 1 and 2), they reversely differentiate into repairing the phenotype cells. From the proximal stump to the distal stump, the Büngner band is formed, which plays a vital role in the peripheral nerve regeneration (18, 19). Furthermore, every Schwann cell has a set of genes. These genes can encode proteins or biologically active substances that promote nerve regeneration and prevent cell apoptosis, such as laminin, type V collagen, and nerve growth factor (NGF). Nerve regeneration is precise. Generally, the speed of nerve regeneration is 3–4 mm/day after compression and 2–3 mm/day after laceration. The regenerated nerve fibers can dominate the muscle preferentially (20).

### Traditional Repairing Strategies

With the development of medical technology and equipment, the treatment of peripheral nerve injury has made significant progress. Various strategies for peripheral nerve repair are summarized here. Briefly, there are two kinds of common repair strategies: surgical repair (such as neurorrhaphy, nerve transfer, nerve grafts, and tubes) and non-surgical repair (such as magnetic and electric field, He–Ne laser, electro photoluminescence, and traditional Chinese medicine) (Table 2) (3, 22).

**Table 2:** Repair strategies of peripheral nerve injury (PNI).

**Repair strategy**	**Name**	**Specific method**	**Features (including advantages and disadvantages)**	**Animal and preclinical studies (References)**
Surgical repair	Neurorrhaphy	First, remove dead tissue, then the nerve suture end to end.	Commonly used in the case of minor damage, the recovery effect depends on the distance from the damaged site to the target organ.	(3)
	Nerve transfer	Connect the proximal nerve stump directly to the muscled abdomen.	Alternative therapies for nerve transplantation reduce proximal nerve damage and a long surgical treatment window (~4 months).	(21)
	Nerve grafts	Take the autologous nerve and transplant it to the nerve defect site.	It is the gold standard for treating long gap nerve injury. Still, it has several disadvantages, such as the limited availability of donor tissue, the sacrifice of functional nerves, and the potential formation of a neuroma.	(22)
	Tubes	Nerve conduits (simulating the physiological structure of healthy nerves, providing physical protection for peripheral nerve regeneration, and stimulating endogenous nerve regeneration potential), essential elements: scaffold, seed cells, cytokines.	The repairability depends on the type of injury, the material of the catheter, etc. It will not damage the self-healthy nerves.	(23)
Non-surgical repair	Magnetic and electric field	Examples include therapeutic ultrasound, low-intensity laser therapy (LLLT), transcutaneous electrical nerve stimulation (TENS), and pulsed electromagnetic field therapy (PEMF).	Adjuvant treatments, various methods have different therapeutic effects. For example, LLLT may be beneficial in patients with rotator cuff disease.	(24)
	He-Ne laser	780 nm laser irradiation treatment.	A specific wavelength of light can improve peripheral nerve function.	(25)
	Electro photoluminescence	Electric acupuncture stimulation down-regulate mir-1b, promote Schwann cell proliferation and nerve repair.	Auxiliary means for peripheral nerve repair.	(26)
	Traditional Chinese medicine	Such as internal and external application, acupuncture, massage, bloodletting, etc.	The methods are diverse and influential. For example, Astragalus (inhibit the expression of caspase-1 and caspase-3, reduce neuronal apoptosis) and some treatment principles are still under study.	(27, 28)

A nerve suture is a standard method for the surgical treatment of nerve injury. The specific process is to remove the necrotic tissue and then suture the nerve end to end. The repair effect depends on the perfection of the repair technology and the distance from the injury site to the target tissue. If the space is too long, even if the nerve stumps are aligned, the time for axons to regenerate and connect to the corresponding part exceeds a time window, which will impair the effect of peripheral nerve repair (3). Nerve suture relies on the axon to produce collateral sprouting. During the operation, part of the axon of the nerve of the donor was artificially destroyed, causing its terminal to germinate, refilling the gap between the nerve stump (29).

A nerve transplantation is another surgical method, although autologous nerve transplantation is the gold standard for treating the long gap nerve injury. In large peripheral nerve lesions, a vascularized nerve transplant can provide blood to the nerve stump, prevent hypoxia, and provide nutrients (3). However, the neuronal process requires a complete nerve donor typically taken from the autologous sensory nerve, which will damage the original healthy tissue (21). With the development of decellularization technology, the allogeneic nerve transplantation can replace the autologous nerve transplantation, which reduces the damage to the autologous nerve and weakens the immune response to achieve the purpose of repair. Zhang et al. completely demyelinated and cellularized the heterogeneous nerves, leaving natural NGF and ECM, integrated them with the autologous nerve-differentiated adipose-derived mesenchymal stem cells (MSCs) to form a nerve conduit. The conduit was implanted in the rats to repair 10 mm sciatic nerve defects (30).

When there is no graft, another intervention may be used, i.e., nerve transfer. The proximal nerve strain is directly connected to the muscled abdomen (22). It can effectively restore the early damage caused by the loss of function, but it requires strict monitoring of the physiological state of the donor nerve by electromyography. The principle is that the healthy nerve bundles will be redirected to the distal part of the injured nerve (near the neuromuscular junction) to replace the role of the original nerve to achieve functional motor recovery. For the PNIs that cannot be regenerated entirely within a time window, nerve transfer helps protect and preserve the distal motor endplates until the natural axons are wholly regenerated (30). Different from the first three methods, the principle of “tubes” repair of nerve injury is to simulate the physiological structure of the healthy nerve, provide physical protection for peripheral nerve regeneration, and stimulate endogenous nerve regeneration potential. The preceding “tubes” were often natural products, such as muscle bundles, amniotic tubes, and venous tubes. The therapeutic effect is unsatisfying.

Over the past few years, the nerve-guided catheters are developed consisting of scaffolding and cells, which have opened a new world for peripheral nerve repair strategy. Of course, the role of non-surgical treatment in the repair of the peripheral nerves is not negligible. In a randomized controlled trial of the patients with incomplete long-term PNI, 780 nm laser irradiation treatment found that a specific wavelength of light can gradually improve the peripheral nerve function, leading to significant functional recovery (25). In addition, electro-photoluminescence has the potential to affect the peripheral nerve regeneration. A study found that after electroacupuncture stimulation of the injured site, mir-1b in the injured local nerve was regulated downward. *In vitro*, it was confirmed that the overexpression of mir-1b could inhibit the expression of brain-derived neurotrophic factor (BDNF) in the rat Schwann cell line. The research may indicate that the electroacupuncture might promote Schwann cell proliferation and recovery of sciatic nerve function by downregulating mir-1b (26).

A traditional Chinese medicine can also reduce neuronal mortality and promote the peripheral nerve regeneration. The treatment methods typically include internal and external application, acupuncture, massage, and bloodletting. The studies have demonstrated that the active principles of the traditional Chinese medicine have effects on anti-apoptosis and neurogenesis (27). Taking the example of Buyang Huanwu decoction, it contains four active ingredients, namely, alkaloid, glycoside, polysaccharide, and aglycone. It was found that they may reduce the production of inflammatory cytokines and reduce the neuronal apoptosis by inhibiting the expression of caspase-1 and caspase-3 (30). As well, Zhong et al. used a preparation of Astragalus (refined from the active ingredients of Astragalus extract) as an inducer and found that it may regulate the differentiation of MSCs into the neuron-like cells through the Wnt signaling pathways. This discovery provides an idea for using the preparation of Astragalus in the treatment of neurodegenerative diseases (28). Li et al. studied that Astragalus polysaccharides can protect mitochondria and anti-aging by inhibiting mitochondrial damage and recovery reactive oxygen species (ROS), meaning that they can be used to repair peripheral nerve damage (31).

### Repair Strategies for Nerve Tissue Engineering

After nerve suture, it took a long time to appreciate the importance of the nerve injury repair strategies and mechanisms. Until medical research entered the microcosmic domain, the neural microenvironment, the assessment of nerve injury, and the cellular and molecular mechanisms involved, and the clinical therapeutic principle for peripheral nerve injury were gradually understood. The methods of nerve tissue engineering for peripheral nerve injuries were also developed.

The core of nerve tissue engineering is to establish the composition of a three-dimensional complex with the cells and biological materials, and the key is manufactured scaffolds, selecting the seeding cells, and adding the neurotrophic factors (32). Compared with other treatment methods, it has taken another route, which develops nerve guidance conduits (NGCs) together with the nerve regeneration cells and small biological molecules to replace the traditional nerve transplantation. The applications of NGCs effectively avoid an immune response, and even in some of the injury scales, the clinical treatment effect is equivalent to the nerve autografts (33, 34).

Nerve repairing with the catheter is a part of the repair of PNI by tissue engineering, while recovering the function of the target organ is another part, especially rehabilitation treatment of muscle atrophy (35). That is because the nerve injury causes double damage. After PNI, muscle denervation causes atrophy and loss of original function.

The strategy of nerve tissue engineering to repair the PNI combines the advantages of the former two methods, changes the original repair ideas, and transforms the so-called “replace the damaged nerve” repair approach into “guide and promote the axon regeneration of the damaged part.” The purpose of nerve tissue engineering is to establish NGCs, which can accurately guide the reconnection of nerve stumps. In some long nerve gaps, it shows nerve recovery ability comparable with the autologous nerve transplantation (32, 35, 36). In the following chapters, the composition, development, and recent research results of NGCs will be described (Table 3). It can be seen from the table that excellent NGCs generally meet the following characteristics: absorbable/degradable, compound SCs/cytokine/cell affinity, topographic clues inside the scaffold conform to cell cord growth, and good elasticity/ductility. These NGCs can adapt to the repair of peripheral nerves with longer gaps (15 mm gap) and effectively promote nerve regeneration. Among them, the NGCs of the composite biomaterials have superior biocompatibility, biodegradability, excellent mechanical properties, good mechanical properties, and cell affinity. They are superior to the natural polymers and non-biosynthetic polymers in repairing the nerves (39, 44).

**Table 3:** Material, design, and applications of currently available nerve guidance conduits (NGCs).

**Material**	**Composition, design**	**Animal, Nerve**	**Injury**	**Outcome**	**References**
Silicone	Non-degradable silicone tube	Rat, sciatic nerve	6 mm gap	The nerve trunk regenerates in the first few weeks, bridging the gap between the proximal and distal stumps. And in each case, the regenerative nerve appears as a cord-like structure surrounded by transparent liquid.	(37)
Collagen	Combination of absorbable collagen catheter and autologous SCs (200,000 cells/μl)	Rat, sciatic nerve	13 mm gap (critical size defect)	After 4 weeks, the addition of Schwann cells can enhance the regeneration of myelinated axons. After 16 weeks, the regeneration effect is similar to the reversed autograft.	(38)
Composite hydrogel	Catheters composed of different volumes of polyacrylamide, graphene oxide, gelatin, and sodium alginate (PAM/GO/Gel/SA, PGGS), inner diameter 2 mm, diameter 6 mm	Rat, sciatic nerve	3 mm gap	The catheter has good elasticity, flexibility, and mechanical properties. HE staining, Masson's trichrome staining, and immunohistochemistry have confirmed that it has a good effect in repairing sciatic nerve injury.	(34)
Poly (L-lactic acid) (PLLA)/soy protein isolate (SPI)	Highly oriented poly (L-lactic acid) (PLLA)/soy protein isolate (SPI) nanofiber nerve catheter	Rat, sciatic nerve	10 mm gap	Three months later, the motor function recovery of the experimental group was better than that of the autograft group. The density of regenerated myelinated nerve fibers in the BMSC-(BDNF + GDNF) group was 16,940.5/mm^2^, higher than the autograft group (16,206.4/mm^2^).	(39)
Collagen, chitosan	Chitosan tubes bind aligned extracellular matrix (proteins and cells)	Rat, sciatic nerve	15 mm gap (critical size defect)	Four months later, SC alignment bracket 15 mm nerve defect that the regeneration success rate was 100%.	(40)
Peripheral epineurium	Embryonic stem cell-derived neural progenitor cells are implanted into the gap between nerve stumps, and the peripheral epineurium serves as a natural conduit.	Rat, sciatic nerve	10 mm gap	Three months after nerve transection, H & E staining examination showed significant apparent transected nerve regeneration and nerve reconnection.	(41)
Heterogenous fibrin	Heterologous fibrin sealant scaffold	Rat, sciatic nerve	5 mm gap	Catwalk and von Frey's functional recovery tests showed the regeneration of sensory fibers and active recovery.	(42)
Poly (ε-caprolactone) (PCL), nanofibers	IL-10 Conjugated electrospun poly (ε-caprolactone) (PCL) nanofiber	Rat, sciatic nerve	10 mm gap	The scaffold can significantly change the phenotype of macrophages in the body and affect peripheral nerve regeneration.	(43)
Hydrogels, nanoparticles	3D printed nerve catheters with an inner diameter of 1.5 mm, an outer diameter of 2.5 mm, and a length of 13 mm	Rat, sciatic nerve	10 mm gap	The nerve in the experimental group recovered well, and the nerve conduction velocity was 30.4 m/s after 3 months, which was not much different from the autograft group (33.9 m/s).	(44)
Human fibroblasts	Bio3D catheter with an inner diameter of 2 mm and a wall thickness of 500 microns	Rat, sciatic nerve	5 mm gap	At 24 weeks after surgery, no tumors were observed in any rats in the Bio3D group. They showed apparent nerve regeneration, which was not significantly different from the autologous nerve transplantation group.	(33)

### Scaffold

Scaffold fabrication is the cornerstone of NGCs. An ideal scaffold should be passive physical support connecting the proximal and distal stump and positively meeting the needs of multiple nerve regeneration. The appropriate scaffolds should provide a place for the growth factors to gather and SCs to grow, protect, and guide the proximal stump to extend to the distal stump, like a bridge to connect the distal nerve (45). At the same time, it can isolate the newborn axons from the external environment, avoid many inflammatory cells infiltrating into the injured area, help accumulate high concentration neurotrophic factors in it, and reduce scar formation and neuroma (46). In addition, the effects of topographic cues, electrical conductivity, and biodegradability on the nerve regeneration cannot be ignored (47). Although there is no ideal technology reported so far, there are many research reports on the design, materials, and fabrication methods of scaffolds (33, 36, 38).

In general, the scaffold should maintain a tubular or linear structure to adapt to the natural bundle structure of axons in biological design. Its primary characteristics include good mechanical properties, well-biocompatibility, biodegradability, and enough permeability (7). The collagen fiber, fibrin, poly (lactic-co-glycolic-acid) (PLGA), polylactic acid or polylactide (PLA), poly (propylene fumarate) (PPF), and agarose can be used as the scaffold materials, and there are pros and cons associated with each process (34).

In 1982, it was reported that the non-degradable silicone tube was used to wrap the severed sciatic nerve of the rats to repair the 6 mm gap *in vivo* system (37). The semipermeable collagen catheter could also repair the PNI *in vitro* (48). However, these scaffolds are unstable and inactive *in vivo*, so they are gradually eliminated. With the progress of materials science, more and more high-quality polymer materials are studied. The new scaffolds can simulate the physicochemical and mechanical properties of the biological tissues, have muscular mechanical strength, and their degradation pattern is based on simple hydrolysis (49).

There was a composite hydrogel catheter used for nerve regeneration, which was made from different volumes of polyacrylamide, graphene oxide, gelatin, and sodium alginate (PAM/GO/Gel/SA, and PGGS). The conduit had good elasticity and good mechanical properties. The catheter was found to have high mechanical stress, which adapted to the unexpected changes of nerve tissue during exercise. It promoted the regeneration of the sciatic nerve in the rats (36).

The agarose-based biomaterials have also achieved success in *in vitro* experiments. It is a polysaccharide extracted from agar found in red algae, which has biocompatibility and non-immunogenicity (50). The template agarose scaffolds made of the fiber bundles enhanced the structural stability and promoted the growth and extension of regenerated axon tissue in the gap of more than 10 mm (51). Chávez-Delgado et al. (52) used a chitosan prosthesis containing neurosteroids (progesterone [PROG] and pregnenolone [PREG]) to bridge the 10 mm gap in the facial nerve of a rabbit. The chitosan scaffold has good biocompatibility and slow degradation rate, and can be used as a guide channel for the axon growth. In addition, the chitosan scaffold can act as an *in-situ* neurosteroid delivery device, a long-term release neurosteroid carrier. After 45 days, the regenerated tissue showed myelinated nerve fibers of different sizes and shapes. The statistical methods showed that there was a significant difference between the experimental group and the control group, and the PROG-loaded chitosan prosthesis produced the best-guided nerve regeneration and recovery. The chitosan scaffolds containing PROG are used to bridge the 10 mm gap in the facial nerves of a rabbit, which can promote the regeneration of the injured peripheral nerves, indicating that the function of damaged nerves may be improved by PROG and other neurotrophic substances (53). A chitosan nerve conduit impregnated with neurosteroid PROG was used to induce regeneration of the sciatic nerve in the adult female dogs repairing a 15 mm defect. The histological analysis and electron microscopy studies showed that the damaged distal nerve segment showed a structure similar to that of a normal nerve (54).

Of course, it is too broad to rely solely on the scaffolds to promote peripheral nerve regeneration. With the development of research on the mechanism of nerve injury and repair, the role of nerve scaffolds in the nerve regeneration has become more diverse. Zhang et al. found that the effect of peripheral nerve regeneration with multiple factors (cells, growth factors, and scaffolds) is higher than that of a single element and can be compared with the effect of autologous nerve transplantation. The benefit of multiple factors is higher than that of a single factor and can be compared with the impact of nerve autografts. The bone mesenchymal stem cells (BMSCs) were introduced into the highly oriented poly (L-lactic acid) (PLLA)/soy protein isolate (SPI) nanofiber nerve conduits as the seed cells to repair the 10-mm sciatic nerve defects in the rats, and showed promising results and superior to the autografts group in some aspects (39).

The fabrication of scaffolds is not only simple stacking or cutting. The most common manufacturing methods include solvent casting (with or without salt leaching), gas foaming (with or without the leaching), phase separation, freeze-drying, and electrospinning (55). In addition, the topographic cues and fillers inside the scaffolds also increase the difficulty of production. Therefore, given these requirements, the traditional process is modified, and some unique manufacturing methods are adopted, such as micro-patterning, injection molding, unidirectional freezing, and electrospinning (9).

### Cell-Based Therapy

The process of nerve regeneration could be promoted by the cells. This is a rather complex and highly coordinated cell–cell interaction process in which the cellular, molecular pathways are not yet clarified (56). Therefore, the effect of the single-cell pathway on the nerve regeneration process is uncertain and may even be harmful. However, from the perspective of cells, the problem will become relatively simple.

In many studies, the scaffolds are combined with the peripheral nerve regeneration cells or cells that produce certain NGFs, and the nerve repair effect is enhanced (Table 4) (38, 40, 41). Berrocal et al. (38) used autologous SCs (200,000 cells/μl) combined with an absorbable collagen catheter, which was applied to the sciatic nerve defect in the rats. The results showed that the combined catheter enhanced the bridge PNIs with the extended segmental defects. Gonzalez-Perez et al. (40) placed MSCs and SCs in the chitosan tubes filled with collagen gel, respectively, which were used to repair a critical size defect of 15 mm in the rat sciatic nerve. And it was found that the SC-aligned scaffolds had the best repair effect. This means that cell technology can be applied to nerve tissue engineering, especially in the long nerve gap, and has a good prospect.

**Table 4:** Commonly used cells in neural tissue engineering.

**Cell**		**Repair mechanism**	**Advantages and disadvantages**	**Applications**	**References**
Schwann cell		Create an environment that supports nerve regeneration; guide the direction of axon growth	The most commonly used, but lack of some characteristics of ideal transplantable cells for tissue engineering, such as easy harvesting, rapid expansion in culture, and low immunogenicity	NGCs filled with SCs can repair long segment gaps faster and more effectively than using tubes alone.	(3, 32, 57)
Stem cell	Embryonic stem cells	Highly undifferentiated; totipotent; the neural phenotype of embryonic stem cells can differentiate into Schwann cells and perform physiological functions after transplantation.	Easy to obtain, rapid culture expansion, prone to tumorigenesis, moral and ethical issues	Using the peripheral nerve membrane as a natural conduit, implanting embryonic stem cell-derived neural progenitor cells can promote the repair of severely damaged peripheral nerves.	(41)
	Neural stem cells	Can differentiate into neurons and Schwann-like cells and secrete various important neurotrophic factors, such as brain-derived neurotrophic factor, fibroblast growth factor, nerve growth factor.	Easy to obtain, rapid culture expansion, prone to tumorigenesis, moral and ethical issues	Intravenous injection of neural stem cells (NSCs) can cause physiological nerve repair, thereby reducing neuropathic pain symptoms.	(58, 59)
	Induced pluripotent stem cells	Terminally differentiated cells return to a pluripotent state or form embryonic stem cell lines under specific conditions.	Abundant sources, easy to obtain, rapid expansion, difficult to cultivate, and prone to tumorigenesis, moral and ethical issues	Implantation of polycaprolactone (PCL) scaffolds loaded with activated Schwann cells (ASCs) and neural stem cells derived from induced pluripotent stem cells can promote the recovery of motor function in rats.	(60)
	Adipose Stem Cell	Differentiate into various phenotypes, such as osteoblasts, chondrocytes, and muscle cells. Neurophenotypic ASCs have neurotrophic characteristics, can differentiate into Schwann cell-like cells, and secrete a variety of neurotrophic factors (nerve growth factor, brain-derived neurotrophic factor)	The most practical, easy to harvest, and has a strong potential for differentiation, moral and ethical issues	ADSCs can improve neuronal differentiation and nerve repair.	(61–63)
Macrophages		M2 type macrophages are activated in a specific microenvironment and participate in anti-inflammatory response and tissue repair; remove axons and myelin fragments in the distal stump to promote axon regeneration.	The function of macrophages can be adjusted by changing the microenvironment, such as up-regulating IL-10 and collagen VI; the use of regulatory factors requires fine control to produce positive therapeutic value.	Electrospinning combined with IL-10 and PCL nanofiber scaffolds can induce the polarization of macrophages to the M2 activation state and participate in nerve repair.	(56)

#### Schwann Cells

Schwann cells are glial cells in the peripheral nervous system and the most used seed cells in nerve tissue engineering. It can be made and repaired together with the scaffolds or used to enhance nerve regeneration separately. It is divided into a myelinated cell and a non-myelinated cell according to its structure and function. The former surrounds thicker axons, while the latter surrounds some slender axons. They can support and nourish nerves, have the ability to repair peripheral nerve damage and promote peripheral nerve regeneration. After the PNI, they reversely differentiate into the progenitor-like cells through genetic reprogramming and further transform into a repair phenotype to participate in the peripheral nerve regeneration (18, 64). The complex process involves the downregulation of the myelinating genes and high expression of regeneration genes, such as the upregulation of transcription factors (e.g., c-Jun) and histone deacetylase 1 and 2 (HDAC1/2) are involved (18, 19).

Schwann cells have two main functions for peripheral nerve repair: creating an environment to support the nerve regeneration and guiding the direction of axon growth. After nerve fiber transection, on the one hand, myelin sheath and axon fragments need to be removed timely and effectively. Otherwise, the efficiency and accuracy of reinnervation will be reduced if the time is too long, which is not conducive to the tissue regeneration (65). In the distal stump, the SCs and macrophages can help axons produce a pro-migration environment by removing the axons and myelin fragments.

On the other hand, if nerve stumps atrophy or nerve defect, the gap will be filled with the inflammatory cells and mediators, peripheral nerve cells, fibroblasts, and ECM, which is called the “bridge” of a new composite tissue reconnection. SCs need to cross the new bridge to guide axon regeneration. It cannot migrate directly into the matrix and depend on the macrophages, which secrete vascular endothelial growth factor A (VEGF-A) and other cytokines. Angiogenesis, remyelination, and axon regeneration occur sequentially in the process of nerve regeneration. A VEGF-A is a critical biological factor in angiogenesis, promoting the development and formation of blood vessels and protecting the motor and sensory neurons (66). Neuromodulin 1, endothelin, and Notch signaling molecules are also involved in this process. Neuromodulin 1 regulates the SC migration and proliferation by binding to ErBb2/3 on SCs. Notch signaling accelerates the SC transformation (67). When the SCs form cord-like structures along the new blood vessels and pass through the new bridge, that can guide the axons to grow in the right direction and promote peripheral nerve regeneration (18, 68). In addition, the effects of cytokines secreted by the SCs on the peripheral nerve regeneration cannot be ignored, such as neurotrophic factors (e.g., GDNF and artemin), adhesion molecules (e.g., N-cadherin and N-CAM). The studies have shown that the SCs can synthesize some neurosteroids (such as, PROG and PREG). These synthetic products can interact with the intracellular receptors to activate the synthesis of some myelin proteins (P0 and PMP22) or act on the gene encoding transcription factors Krox-20 (Egr-2) to induce myelination (52).

Many studies have shown that there are many difficulties in using the NGCs to repair the peripheral nerve (3, 32, 57). For example, the effect of repair *in vivo* is poor, and some severe injuries cannot be fully repaired. This may be since nerve conduits can only provide physical support but cannot give endogenous or exogenous regenerative power. Therefore, simple nerve conduits are only suitable for supporting the growth of the nerve cells and transporting nutrients, and cell therapy is an essential basis for repairing the PNI. The first crucial step to introduce the SCs based therapy into the PNI is to add SCs to the guiding catheter. It is proved that the NGCs filled with SCs can repair the long segmental spaces faster and more effectively than the tubes alone (38). In addition, in some studies, SCs were programmed to overexpress neurogenic factors, such as VEGF-A, and then loaded onto the inner wall of hydroxyethyl cellulose/soy protein isolate/polyaniline sponge (HSPS) conduits and transplanted to the damaged site. Three months later, immunofluorescence co-staining showed that MBP + Schwann cells in the HSPS-SC (VEGF) group were far superior to the HSPS-SC group (MBP protein is a biomarker of Schwann cell myelin differentiation). The final results also showed that the NGCs have good functional and morphological repairs to peripheral nerve injuries (66, 69).

#### Stem Cells

Because of the critical role of endogenous regeneration in the PNI, the SCs are often used as transplantable cells for nerve repair, which has been proved by *in vivo* and *in vitro* experiments. Moreover, due to the limited proliferation ability of SCs, their application is minimal. The self-renewing ability and pluripotency of stem cells are considered the ideal source of seed cells in nerve tissue engineering. Therefore, the scientists hope to repair nerve injury by replacing the cells (35).

Unlike SCs, the stem cells are abundant in variety and source. Nerve tissue engineering needs to be screened from many stem cells. The cells need to include the following characteristics: easy to obtain, rapid expansion in culture, survival in the damaged site, stable transfection and expression of foreign genes, and binding with NGCs (70). The embryonic stem cells (ESCs), neural stem cells (NSCs), induced pluripotent stem cells (IPSCs), mesenchymal stem cells (MSCs), adipose-derived stem cells (ASCs), neural crest stem cells, dental pulp stem cells, skin, and umbilical cord-derived those can be used to repair the PNI (35, 70).

The ESCs are highly undifferentiated cells with totipotency, differentiating into all the tissues and organs in the adult animals. The ESCs can be induced into a neural phenotype before transplantation and can differentiate into the SCs and play their physiological functions after transplantation, promoting angiogenesis, nerve growth, and myelination. Compared with the SCs, the proliferation of ESCs is more active. Moreover, the SCs differentiated by the ESCs can express relevant markers, glial fibrillary acidic protein, S100, and p75, and induce neuronal myelination (71). In the rat sciatic nerve transection model, using a peripheral nerve adventitia as a natural conduit, implantation of the neural progenitor cells derived from the ESCs can promote the repair of severely injured peripheral nerve (41).

The NSCs are the primitive cells in the nervous system and are also the essential cellular sources of the neurons and glial cells. They can be used as an essential cell source for the nerve regeneration. A study showed that after implantation of the NSCs, the abundance of IL12p80 will increase, which directly stimulates SC differentiation and promotes the peripheral nerve recovery (72). The NSCs combined with the NGCs can differentiate into the neurons and Schwann-like cells and secrete many important neurotrophic factors, such as brain-derived neurotrophic factor, fibroblast growth factor, NGF, insulin-like growth factor, and hepatocyte growth factor, after transplanting to the injured area, to promote nerve regeneration (58).

In addition, intravenous injection of the NSCs can cause physiological nerve repair, thus reducing the neuropathic pain symptoms (59). IPSC has always been a research hotspot, which refers to the recovery of terminally differentiated cells to totipotent state or the formation of ESC lines under the specific conditions. In relevant studies, the implantation of polycaprolactone (PCL) scaffolds loaded with active Schwann cells (ASCs) and IPSC -derived NSCs at the injured site can improve the motor recovery of the rats (60). Bone marrow MSCs are pluripotent adult stem cells obtained by bone marrow aspiration or artificial culture. The rat bone marrow MSCs are used as the supporting cells introduced into the silk fibroin (SF)-based scaffolds. After catheter transplantation, it was found that the gene expression of S100 and several growth factors (brain-derived neurotrophic factor, ciliary neurotrophic factor, and fibroblast growth factor) were upregulated, and many ECM components were secreted, such as collagen, fibronectin, and laminin promote the histological and functional recovery of the damaged sciatic nerve in the rats (73). The chitosan/ PLGA nerve scaffolds containing autologous MSCs have been used to repair the long nerve gaps in the large animals (such as bridging the 50-mm-long gap in the sciatic nerve of a dog) (74).

At present, the ASCs are the most valuable source of the transplanted cells (57). The ASCs can be separated from human subcutaneous fat by conventional liposuction under anesthesia and cannot be used directly to repair the nerve damage. They need to be further separated from the vascular matrix component. Otherwise, it will block the nerve conduit and hinder regeneration (59). They are easy to harvest and have strong differentiation potential. They can differentiate into various phenotypes along the mesoderm lineage, such as osteoblasts, chondrocytes, and muscle cells. The neural phenotypic differentiation of ASCs involves a combination of multiple growth factors. The ECM molecules affect the cell viability, adhesion, and neurotrophic behavior of differentiated ASCs, and coating them with the nerve ducts can increase the regeneration rate. Recent studies have shown that human platelet lysates have neurotrophic properties (such as the release of BDNF). The synergistic effect with laminin can enhance the neurotrophic effect of ASCs on the primary neurons *in vitro* (75). The acquisition site of ASCs (the best subcutaneous and perennial) will also affect the differentiation results (76). The neural phenotype ASCs have neurotrophic characteristics and express SC markers (such as glial fibrillary acidic protein (GFAP), S100, and the low-affinity receptor for NGF p. 75), which can differentiate into the SC -like cells and secrete a variety of neurotrophic factors. Several studies have shown that the ADSCs can improve the neuronal differentiation and neural repair (58, 59). Recent studies have found that the exogenous neurotrophic factors can be used to enhance the neurotrophic capacity of the human adipose derived stem cells *in vitro* (66).

In neural tissue engineering, the use of stem cells requires selecting the appropriate type, optimizing the number and methods of transplantation, and using the exogenous factors to ensure cell survival, reduce tumorigenicity, ensure safety, and maximize therapeutic efficacy (70). The human embryonic stem cells (HESCs) overexpressing the fibroblast growth factor 2 (FGF2) combined with heterogenous fibrin sealant scaffold can support the survival and regeneration of neurons in a mouse model of sciatic nerve injury (42). Beyond that, incorporating the ESCs, NSCs, bone marrow MSCs, adipose-derived cells, skin-derived precursor stem cells, and IPSCs enhances the therapeutic effect of tissue-engineered nerve graft (58).

#### Macrophages

The macrophages are mononuclear phagocytes, which show significant plasticity *in vivo* and *in vitro*. As the prominent role of inflammatory cells, there are apparent morphological and functional differences in the different micro-environments. At present, it can be divided into two phenotypes, M1 and M2 macrophages. The M1 macrophages are effectors and induce cells in inflammation and participate in a positive immune response by secreting inflammatory cytokines and chemokines, which activation is related to the lipopolysaccharide (LPS) and interferon-γ (IFN-γ) and so on. The M2 macrophages have a weak ability to antigen presentation, secrete anti-inflammatory cytokines, participate in anti-inflammatory response, and are activated in the specific micro-environments to play the role of immune regulation and participate in tissue repair.

After PNI, it will lead to a local inflammatory reaction, a large number of inflammatory factors are released. The macrophages are recruited by chemokines (CCL2) and gather in the area with other inflammatory cells, participate in Wallerian degeneration of the nerve cells, polarize into anti-inflammatory phenotype (M2) under the effect of IL-4, and participate in axonal regeneration. The results show that the SCs and fibroblasts control and modify the macrophage reactions. And the cytokines secreted by them release into the blood, chemotactic monocytes, and change their behavior (56). In addition, the macrophages can facilitate axon regeneration by removing the axon and myelin fragments in distal stumps (10).

The regulation of macrophage function is an effective strategy to improve the peripheral nerve regeneration and repair injury. Currently, it is also included in the nerve tissue engineering to repair the nerve injuries. Jason R Potas et al. used electrospinning combined IL-10 with the PCL nanofibrous scaffolds to successfully induce the macrophages to polarize to the M2 activated state in the scaffolds and adjacent tissues around the nerve, which confirmed that macrophage function could be regulated by manipulating the cell micro-environment, thus affecting the process of nerve regeneration (43). Recent studies have shown that the macrophages polarize into the M2 type in the micro-environment after peripheral nerve injury, which may involve the IL-10 and collagen VI upregulated. Therefore, IL-4 and other factors can be embedded in the nerve guide catheter to promote the transformation of the macrophage M2 phenotype (11). However, the factors that promote the M2 polarization of macrophages may have the property of promoting tumor growth. Therefore, the use of these factors needs fine control to produce positive therapeutic value for the PNI (43). For example, through cell genetic engineering, some cells overexpressing the above factors are loaded on the nerve scaffolds, which can effectively regulate their release. Or changing the physical or chemical properties of scaffolds, and using additive manufacturing technology to construct the biological components of scaffolds layer by layer, so that the factors can be degraded and released at an appropriate time to achieve the purpose of peripheral nerve repair (3).

### 3D Bioprinting in Nerve Injury Repair

A new technique, 3D printing, is also called additive manufacturing, which means adding materials to make things. 3D printing technology was used in the industrial production before, but now bioprinting is common, such as bioprinting of NGCs (77). Micropatterning, injection molding, and unidirectional freezing are the unique manufacturing methods of some NGCs (9). In recent years, 3D printing has become a matured technique, and its most commonly used technologies include extrusion (fuse manufacturing and direct ink writing), powder, and photoinduced polymerization (77). 3D bioprinting is used to make the biomaterials containing cells, that is, to build the complex 3D functional living tissues or artificial organs (49).

Among them, the droplet-based bioprinting (DBB) can print tissue or organs embedded in the cells based on or without a scaffold. It has been reported that in the rat model, the effect of a 3D printed human fibroblast catheter in repairing a 5 mm nerve gap is not significantly different from that in autologous nerve transplantation (33). Because of the personalized manufacturing characteristics of 3D printing, it is possible to customize the size, shape, and structure required. The scaffolds printed combine with biological factors (such as nanoparticles and cells) to achieve exact control (77). Therefore, the NGCs made of various biomaterials are produced through research. Recently, a catheter is developed, consisting of nanoparticles in hydrogel and hydrogel matrix. The hydrogel provided a physical microenvironment for axonal elongation, and the nanoparticles continuously released drugs to promote the nerve regeneration. The drug is an inhibitor of the Hippo pathway. The previous studies have found that the Hippo pathway affects the peripheral nerve regeneration (44). Therefore, Jie Tao et al. upregulated the downstream gene (yes-associated-protein) by targeting the inhibition of this pathway in the SCs to promote the regeneration of PNI. The experimental results showed that the functional nerve conduit could effectively induce the recovery of sciatic nerve injury (78).

### Muscle Tissue Engineering

The target organs of peripheral innervation include the sensory and motor organs (muscles). After PNI, the sensation will remain for several years, even for a long time to repair, and the function can be restored. In contrast, the denervated muscle progressively loses its ability to become reinnervated (35). In the process of muscle atrophy, the proteasome pathway and the autophagy-lysosomal pathway are activated, leading to different amount of loss of muscle mass and loss of its original function (79).

In neurogenic atrophy, it is proved that the regulation of myogenin is a regulator of muscle development and an inducer of neurogenic atrophy. After denervation, myogenin is upregulated, the expression of E3 ubiquitin ligases is decreased, and the muscle proteolysis and atrophy are promoted (80). A study reported that when the PNI was treated early, the atrophy of the distal muscle would stop, and the function would recover (35).

At present, in addition to exercise, there is no effective treatment for reducing the muscle atrophy. Jin Li et al. demonstrated for the first time that CRISPR-based genome editing can specifically target mir-29b in the mouse denervation model, thus preventing angiotensin-II (Ang-II) and myocyte apoptosis induced by Ang-II (81). Beyond that, intramuscular injection of various growth factors (such as IGF-1) and stem cells (such as muscle satellite cells) can also alleviate the muscle atrophy, but its mechanism is still unknown (35).

Additionally, laser phototherapy has potential therapeutic value. The 780 nm laser phototherapy could temporarily retain the function of denervating muscle and accelerate and enhance the axon growth and regeneration after the PNI or reconstruction surgery (25). However, tissue engineering may be a good choice when needed to reconstruct the muscle, and the regenerated axons cannot be accepted after extensive denervation.

The peripheral nerve regeneration strategy includes the treatment of muscle reinnervation. After the PNI, the innervated muscles will atrophy, and muscle regeneration depends on its endogenous regeneration ability (35). The satellite cells, a group of undifferentiated muscle cells located between the basal layer and the plasma membrane of the muscle fibers, are the main participants in muscle regeneration (79). But when the ability is exhausted, the muscle tissue engineering therapy is essential. However, up to now, muscle tissue engineering has not been able to restore all the functions of the replaced muscle (82). The reason might be that the mechanical stress stimulation of the human muscle bundle is challenging to simulate fully. Therefore, the bioreactor can be used to stimulate the cells to achieve muscle tissue reconstruction *in vivo*.

However, the above conditions need to be met in nerve tissue engineering because the loose cells cannot regenerate directly, and scaffold support is also required. Perez-Puyana et al. used electrospinning technology to add elastin to the PCL-based scaffolds for skeletal muscle. The scaffolds had more petite size and better hydrophilicity, and the biocompatibility was also improved (83).

## Summary and Future Prospects

This review describes the pathophysiology of PNI and its repair strategies. The repair strategies involve the intersection of biology, medicine, materials science, and engineering. Neural tissue engineering has proposed many new approaches to manufacture the highly adjustable and functional scaffolds, which have great potential for repairing the PNIs. However, so far, a large number of NGCs developed, whether *in vivo* or *in vitro* experiments, still have a particular gap with autologous nerve transplantation. In the follow-up research, the physical, chemical. and mechanical properties of scaffolds need to be adjusted to optimize the interaction between the cells in the scaffold and the implanted tissue to realize the large-scale promotion and application of NGCs. The biodegradation rate of the scaffold also needs to be controlled to maintain its integrity until the regenerated tissue matures. In addition, the production technology of NGCs is also a hot spot of current research. The 3D bioprinted NGCs support the migration and proliferation of the seed cells. 3D printing based on digital light processing (DLP) technology can develop a more precise internal structure of the scaffold and promote more efficient nerve regeneration.

Given the severity and complexity of the PNI, the current tissue engineering repair strategy still has some problems to be solved. For example, what is the optimal time window for nerve guide catheter implantation? Is it possible to use clinical interventional therapy to reduce the secondary damage to the injured site by implant surgery? In the above, the delivery of the nerve cells, NGF, and drugs can effectively promote axon regeneration, but the interaction of too many cytokines may cause adverse solid immune reactions. Therefore, the study of single-factor NGCs can become a future research hotspot. Vascularization is an essential factor in the survival and function of nerve grafts. The fascial flaps or vascularized nerve grafts have limited clinical applicability and therapeutic effects. The NGCs carrying angiogenic active substances (such as VEGF) is one of the focuses of current research and may become the development direction of NGCs in the future. In addition, a variety of nerve guiding catheters should be designed, such as multicellular components, pro-angiogenesis, and Büngner-like structures to improve the long-distance growth of axons. In the future, the functionalized NGCs produced by 4D printing will be one of the hot spots in the future research on the peripheral nerve regeneration.

## Author Contributions

YL: methodology, writing an original draft, and visualization. ZM: conceptualization, methodology, validation, investigation, writing-review and editing, and funding acquisition. YR and DL: writing an original draft and writing-review and editing. WL: writing original draft. TL: investigation and writing-review and editing. JW: conceptualization, validation, and investigation. HM: conceptualization, validation, investigation, writing-review and editing, and supervision. JZ: validation, investigation, supervision, project administration, and funding acquisition. All authors contributed to the article and approved the submitted version.

## Funding

This work was financially supported by the Shanghai Leading Talents Program in 2020 (No. 110), the National Key R&D Program of China (2020YFC2008700/2018YFA0703000), and the Project of Shanghai Jiading National Health and Family Planning Commission (KYXM 2018-KY-03).

## Conflict of Interest

The authors declare that the research was conducted in the absence of any commercial or financial relationships that could be construed as a potential conflict of interest.

## Publisher's Note

All claims expressed in this article are solely those of the authors and do not necessarily represent those of their affiliated organizations, or those of the publisher, the editors and the reviewers. Any product that may be evaluated in this article, or claim that may be made by its manufacturer, is not guaranteed or endorsed by the publisher.
